# Biochemical characterisation of a novel broad pH spectrum subtilisin from *Fictibacillus arsenicus* DSM 15822^T^

**DOI:** 10.1002/2211-5463.13701

**Published:** 2023-09-07

**Authors:** Fabian Falkenberg, Sophie Kohn, Michael Bott, Johannes Bongaerts, Petra Siegert

**Affiliations:** ^1^ Institute of Nano‐ and Biotechnologies Aachen University of Applied Sciences Jülich Germany; ^2^ Institute of Bio‐ and Geosciences, IBG‐1: Biotechnology Forschungszentrum Jülich Germany

**Keywords:** *Bacillaceae*, biotechnological application, broad pH spectrum, subtilases, subtilisin

## Abstract

Subtilisins from microbial sources, especially from the *Bacillaceae* family, are of particular interest for biotechnological applications and serve the currently growing enzyme market as efficient and novel biocatalysts. Biotechnological applications include use in detergents, cosmetics, leather processing, wastewater treatment and pharmaceuticals. To identify a possible candidate for the enzyme market, here we cloned the gene of the subtilisin SPFA from *Fictibacillus arsenicus* DSM 15822^T^ (obtained through a data mining‐based search) and expressed it in *Bacillus subtilis* DB104. After production and purification, the protease showed a molecular mass of 27.57 kDa and a pI of 5.8. SPFA displayed hydrolytic activity at a temperature optimum of 80 °C and a very broad pH optimum between 8.5 and 11.5, with high activity up to pH 12.5. SPFA displayed no NaCl dependence but a high NaCl tolerance, with decreasing activity up to concentrations of 5 m NaCl. The stability enhanced with increasing NaCl concentration. Based on its substrate preference for 10 synthetic peptide 4‐nitroanilide substrates with three or four amino acids and its phylogenetic classification, SPFA can be assigned to the subgroup of true subtilisins. Moreover, SPFA exhibited high tolerance to 5% (w/v) SDS and 5% H_2_O_2_ (v/v). The biochemical properties of SPFA, especially its tolerance of remarkably high pH, SDS and H_2_O_2_, suggest it has potential for biotechnological applications.

## Graphical abstract

Microbial subtilisins, particularly from the *Bacillaceae* family, have significant potential for biotechnological applications in the enzyme market. In this study, the gene of the subtilisin SPFA from *Fictibacillus arsenicus* was cloned and expressed in *Bacillus subtilis* DB104. SPFA exhibited a very broad pH optimum (8.5–11.5) and high stability in the presence of 5% (w/v) SDS and 5% (v/v) H_2_O_2._

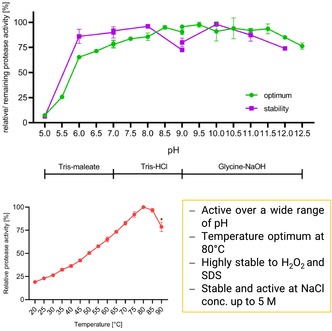

**Abbreviations**
aa: amino acid
*aprE*
: extracellular alkaline protease gene
bp: base pairs
EDTA: ethylenediaminetetraacetic acid
HEPES: 4‐(2‐hydroxyethyl)‐1‐piperazineethanesulfonic acid
IEF: isoelectric focussing
LB: lysogeny broth
LM: length marker
MALDI‐TOF‐MS: matrix‐assisted laser desorption ionisation—time‐of‐flight mass spectrometry
MSA: multiple sequence alignment
PAGE: polyacrylamide gel electrophoresis
PCR: polymerase chain reaction
PDB: protein data bank
pI: isoelectric point
PIS: phylogenetically intermediate subtilisins
PMSF: phenylmethylsulfonyl fluoride
pNA: para‐nitroanilide
SDS: sodium dodecyl sulphate
SPFA: subtilisin protease *F. arsenicus*
suc: N‐succinyl
*T*_m_
: melting temperature

Hydrolytic enzymes produced by microorganisms play a crucial role in a variety of industrial applications due to their commercial value. Here, the largest market share is accounted for by subtilisins or alkaline proteases from microbial sources, particularly from the *Bacillaceae* family [1, 2]. Subtilisins are a group of subtilases classified in the MEROPS database as S8, one of the largest families of serine peptidases [3]. Subtilisins can be further structured into true subtilisins, phylogenetically intermediate subtilisins (PIS), high‐alkaline subtilisins and intracellular subtilisins, among others [4, 5, 6]. In Bacilli and related bacteria, intracellular subtilisins form the main component of the degradome, while extracellular subtilisins primarily contribute to nutritional provisioning due to their broad substrate specificity [4, 7, 8]. In particular, the genus *Bacillus* has been recognised as a valuable source of alkaline proteases such as subtilisin Carlsberg, Savinase, BPN’ and PB92, which are mainly used as active ingredients in detergents due to their properties, such as good performance against proteinaceous stains, high stability towards components of the detergent matrix and high operating temperatures [9, 10]. In addition, they are used in industrial applications such as food processing, wastewater treatment and cosmetics instead of aggressive chemicals [9, 11, 12]. Because of their industrial importance, subtilisins have been intensively studied for their biological function in order to understand the mechanism of catalysis and the structure–function relationship [13]. An extracellular subtilisin typically has a molecular weight of about 27 kDa, consists of a signal peptide, propeptide and a peptidase domain and is secreted as a precursor of about 39 kDa [14, 15, 16].

Nature holds with its almost unlimited microbial biodiversity great potential for enriching the repertoire of known and new enzymes [17]. However, the classical approach of isolation and cultivation of microorganisms from a specific environment with the extraction of genomic DNA or the purification of proteins and gene identification is a time and labour‐intensive procedure [18]. The increasing number of genomic or metagenomic sequencing projects, which provide an exponentially growing amount of data on uncharacterised proteins due to automatic annotation, offers an alternative source for finding new protease genes for industrial applications [19]. We recently analysed this unused information and conducted a search for new uncharacterised subtilisins from the *Bacillaceae* family [6]. These sequences were phylogenetically categorised into the subgroups true subtilisins, PIS and high‐alkaline subtilisins. Here, we selected a sequence from the phylogenetic tree of true subtilisins obtained from *Fictibacillus arsenicus* DSM 15822^T^ for biochemical characterisation, as the sequence originates from a yet unexplored sequence section. *F. arsenicus* is a Gram‐positive, endospore‐forming and arsenic‐resistant bacterium, which was isolated from a concretion of arsenic ore obtained from a borehole in India by Shivaji *et al*. [20]. The strain grows optimally at a temperature of 30 °C and a pH of 7.0, while also tolerating up to 1.0% NaCl. The bacterium was initially classified as *Bacillus arsenicus* in 2005 and then reclassified as *Fictibacillus arsenicus* [21].

Research into more robust subtilisins with polyextremotolerant properties is not only scientifically crucial to better understand the mechanism of enzyme adaptation but is also of huge practical importance for the development of proteolytic biocatalysts with enhanced versatility in relation to different extreme conditions that are often encountered in industrial applications. Here, we report on the gene for subtilisin WP_077360649 from *Fictibacillus arsenicus* DSM 15822^T^, which was cloned, overexpressed in *B. subtilis* DB104 and purified by ion‐exchange chromatography. This is the first report on the biochemical characterisation of a recombinant subtilisin protease from *F. arsenicus* (SPFA) and for the first time from the genus *Fictibacillus*.

## Material and methods

### Bacterial strains and growth conditions

*Fictibacillus arsenicus* DSM 15822^T^ was purchased from the German Collection of Microorganisms and Cell Cultures GmbH (DSMZ) and cultivated in medium 220 with 10 mg·L^−1^ MnSO_4_ at a temperature of 30 °C as recommended by the DSMZ. The InnuSPEED Bacteria/Fungi DNA Kit (Analytik Jena™, Jena, Germany) was used for genomic DNA preparation from an overnight culture. *Bacillus subtilis* DB104 was used for cloning and protein production and cultivated in LB medium (Luria/Miller; Carl Roth, Karlsruhe, Germany) at 37 °C [22].

### Plasmid construction/cloning and bioinformatic analysis

For recombinant protease production with *B. subtilis* DB104, pFF‐RED, a pBC16‐based expression plasmid (Acct. No. U32369.1) [23], was used as previously described [24]. The DNA sequence encoding the gene for SPFA, including the signal peptide, the propeptide and the peptidase domain, was amplified from the genomic DNA in a polymerase chain reaction (PCR) using the Phusion® Hot Start II high‐fidelity polymerase (Thermo Fisher Scientific GmbH, Karlsruhe, Germany) according to the manufacturer's recommendations. The NCBI reference sequence NZ_MQMF01000001.1 was used to design primers for the *aprE* gene (extracellular alkaline protease) of WP_077360649 [25, 26]. The oligonucleotides for PCR were obtained from Eurofins Genomics GmbH (Ebersberg, Germany; Table S1). Cloning and transformation were performed as previously described [27]. Bioinformatic analysis of the amino acid sequence, homology modelling and multiple sequence alignment (MSA) as previously reported [27].

### Recombinant protease production and purification

A 1‐L scale DASGIP® parallel reactor system (DASGIP, Jülich, Germany) was used for the production of SPFA by *Bacillus subtilis* DB104 as previously described [24]. Successful production was confirmed by SDS/PAGE (sodium dodecyl sulphate–polyacrylamide gel electrophoresis) and by a proteolytic activity assay using suc‐AAPF‐pNA (*N*‐succinyl‐Ala‐Ala‐Pro‐Phe‐p‐nitroanilide) and azocasein as substrate. Protease purification was performed as previously detailed [24]. For SPFA, an anion exchanger (25 mL Q‐Sepharose FF, GE Healthcare, Chicago, IL, USA) and a pH of 8.0 for running (10 mm HEPES (4‐(2‐hydroxyethyl)‐1‐piperazineethanesulfonic acid)‐NaOH buffer) and elution buffer (10 mm HEPES‐NaOH, 1 m NaCl) was used.

### Enzyme activity assay

The determination of hydrolytic activity was performed by using the tetrapeptide substrate suc‐AAPF‐pNA (BACHEM, Bubendorf, Switzerland) at 30 °C in 100 mm Tris–HCl buffer, pH 8.6, containing 0.1% (w/v) Brij®35 as described elsewhere [24, 28]. Azocasein (Sigma‐Aldrich, Schnellendorf, Germany) was used as a more complex substrate at 37 °C in 100 mm Tris–HCl buffer, pH 8.6, as previously described [24, 29]. In addition, various synthetic 4‐nitroanilide substrates were used to determine the substrate specificity of SPFA as previously reported [24].

### Protein electrophoresis, measurement and analytical methods

Protein concentration was quantified using Roti®Nanoquant (Carl Roth) with bovine serum albumin fraction V (Carl Roth) as reference according to the manufacturer's recommendations. The molecular mass of the purified SPFA was analysed by SDS/PAGE and matrix‐assisted laser desorption ionisation time‐of‐flight mass spectrometry (MALDI‐TOF‐MS), as previously described [24]. For the determination of the isoelectric point (pI), purified SPFA was rebuffered in 10 mm HEPES‐NaOH pH 8.0 using centrifugal spin columns (VWR, Radnor, PA, USA) with a molecular mass cut‐off of 3 kDa. Isoelectric focussing–polyacrylamide gel electrophoresis (IEF‐PAGE) was performed using an IEF SERVALYT™ PRECOTES™ 3–10 gel (SERVA, Heidelberg, Germany) according to the manufacturer's recommendations.

### Effects of pH and temperature

The pH optimum was determined in 0.1 m Tris‐maleate buffer (pH 5.0–7.0), 0.1 m Tris–HCl (pH 7.0–9.0) and 0.1 m glycine‐NaOH (pH 9.0–12.5) at 30 °C using the suc‐AAPF‐pNA assay. The stability of SPFA at different pH levels was assayed by preincubating SPFA in said buffers for 24 h at 4 °C and residual activities were measured under standard reaction conditions for the suc‐AAPF‐pNA assay. For the determination of the melting point, the fluorescent dye SYPRO™ Orange (Thermo Fisher Scientific GmbH) was used in the thermal shift assay as previously described [24]. Temperature stability was investigated by measuring the residual activity after incubation of SPFA in 10 mm HEPES‐NaOH, pH 8.0, at 20 and 50 °C for 3 h, as previously detailed [24].

### Effects of different additives

To test the impact of additives, 1 and 5% (w/v) SDS and 1 and 5% (v/v) H_2_O_2_ were added to the enzyme solution and incubated for 1 h at 10 °C in 10 mm HEPES‐NaOH, pH 8.0. The inhibition of SPFA with PMSF was investigated by incubating SPFA in 10 mm HEPES‐NaOH with 1 mm PMSF, pH 8.0, for 30 min on ice. Standard suc‐AAPF‐pNA activity assay was used to determine the residual activity. To investigate the influence of NaCl (0–5 m) on SPFA, hydrolytic activity was measured using the above‐described suc‐AAPF‐pNA assay with the addition of NaCl in the reaction buffer as previously reported [24]. In addition, stability at different NaCl concentrations (0–5 m) was investigated by incubating SPFA in 10 mm HEPES‐NaOH, pH 8.0, at 20 °C for 2 h. The effect of ethylenediaminetetraacetic acid (EDTA) was investigated by incubating the protease for 12 h at 4 °C with 20 mm EDTA as previously described [24]. The influence of different metal ions was investigated by incubating the protease with 1 mm each of MgSO_4_, ZnCl_2_, MnCl_2_, CaCl_2_, CoCl_2_, NiSO_4_, FeSO_4_ and CuSO_4_ in 10 mm HEPES for 1 h at room temperature. Standard suc‐AAPF‐pNA activity assay was used to determine the residual activity.

## Results

### Cloning and expression of *aprE_F. arsenicus* in *B. subtilis* DB104

The uncharacterised protease SPFA was chosen from our previous report on a data mining approach screen for new subtilisins from *Bacillaceae*, as it displays a more distant sequence homology to known subtilisins [6]. The coding sequence of aprE_*F. arsenicus* for the protease SPFA, including the signal peptide, the propeptide and the peptidase domain, was amplified, and a fragment of 1147 bp was obtained. The PCR product was cloned into the vector pFF‐RED. The transformation of *B. subtilis* DB014 was verified by the emerging clearing zones on LB agar plates with 2.5% (w/v) skim milk around the colonies and analysed by plasmid preparation and restriction analysis. Sequence data obtained by Sanger sequencing confirmed that it was identical to the genomic nucleotide sequence of the *aprE_F. arsenicus* gene available in GenBank.

### Bioinformatic analysis

**Fig. 1:**
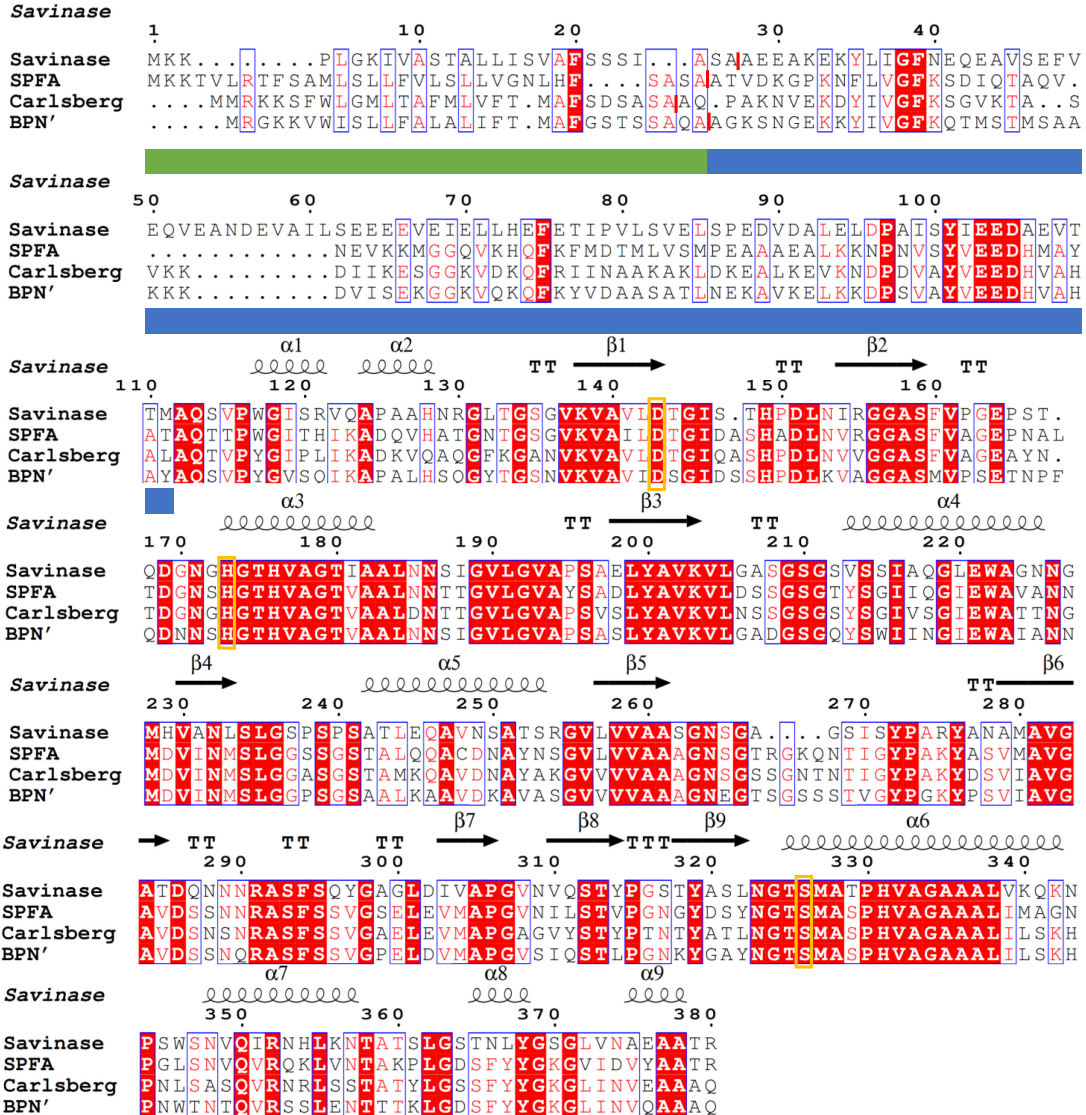
Multiple sequence alignment (MSA) of SPFA with Savinase (WP_094423791.1), subtilisin Carlsberg (WP_020450819.1) and BPN’ (WP_013351733.1). clustal omega was used for the alignment [59]. ESPript 3.0 with Savinase (PDB: 1C9J) as a template was used to analyse the MSA [60]. Signal peptide sequence (green bars); propeptide (blue bars) of SPFA. Red bars indicate individual signal peptide cleavage sites. Secondary structure elements: helices with squiggles, β‐strands with arrows and turns with TT letters. The catalytic triad (Asp^143^, His^173^, Ser^326^; Savinase numbering) is marked with orange boxes.

The *aprE* gene from *Fictibacillus arsenicus* DSM 15822^T^ comprises 1140 bp encoding a protein of 380 amino acids (aa). The signal peptide prediction revealed the presence of a Sec signal peptide with a probability above 99%. Using MSAs, the propeptide was identified and is labelled in Fig. 1. An S8 peptidase domain containing 275 amino acids, a 32‐aa signal peptide and a 73‐aa propeptide were identified. The *in silico* analysis of mature SPFA showed a molecular mass of 27.57 kDa and a pI of 5.8. The catalytic triad consists of Asp^32^, His^64^ and Ser^221^ (numbers based on the mature protease sequence). The analysis of our previously reported phylogenetic tree with well‐characterised proteases of the three subtilisin families (true, high‐alkaline, phylogenetically intermediate) retrieved from MEROPS [3] and the UniProt database [30], as well as data mined sequences, demonstrates that SPFA is clearly a member of the true subtilisin subgroup [6].

**Fig. 2:**
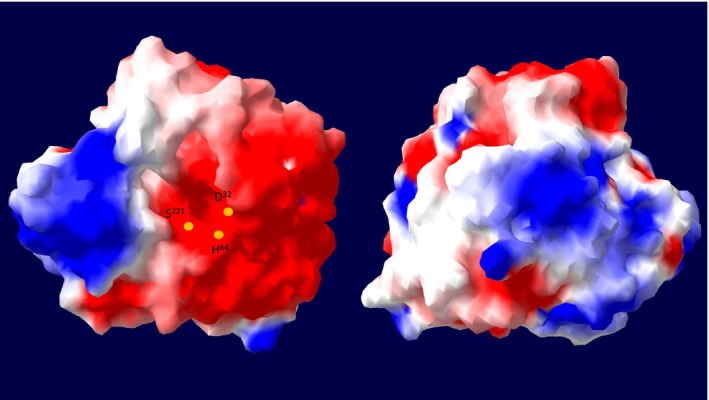
Structural model of SPFA with its calculated surface electrostatic potential. (Left) top view of the active site; (right) rear view to the active site. Swiss‐PdbViewer was used to calculate the electrostatic potential at pH 7.0 and negative charges (red), positive (blue) and neutral (white) are shown.

A homology modelling‐based structural analysis with the I‐TASSER server was performed with the peptidase unit of SPFA, yielding a model with a C‐score of 1.55 (Fig. S1). The C‐score ranges from −5 to 2 and higher values indicate higher confidence of the model [31]. A high template modelling (TM) score is given for BPN’ (PDB: 1S01) of 0.997, where a TM value of 1 specifies a perfect correlation for two structures [32]. The metal‐binding prediction suggested two Ca^2+^‐binding sites involving the side chains Gln^2^ and Asp^41^ and several side chains of the loop‐forming residues 75–81 for the first Ca^2+^‐binding site. The side chains of Ala^169^, Tyr^171^ and Val^174^ are involved in the second Ca^2+^‐binding site. In addition, the homology model was used to calculate the electrostatic potential at pH 7.0, as shown in Fig. 2. SPFA is mainly negatively charged around the active site, while neutral, positive and negative charges are balanced on the backside.

### Protease production and purification

**Fig. 3:**
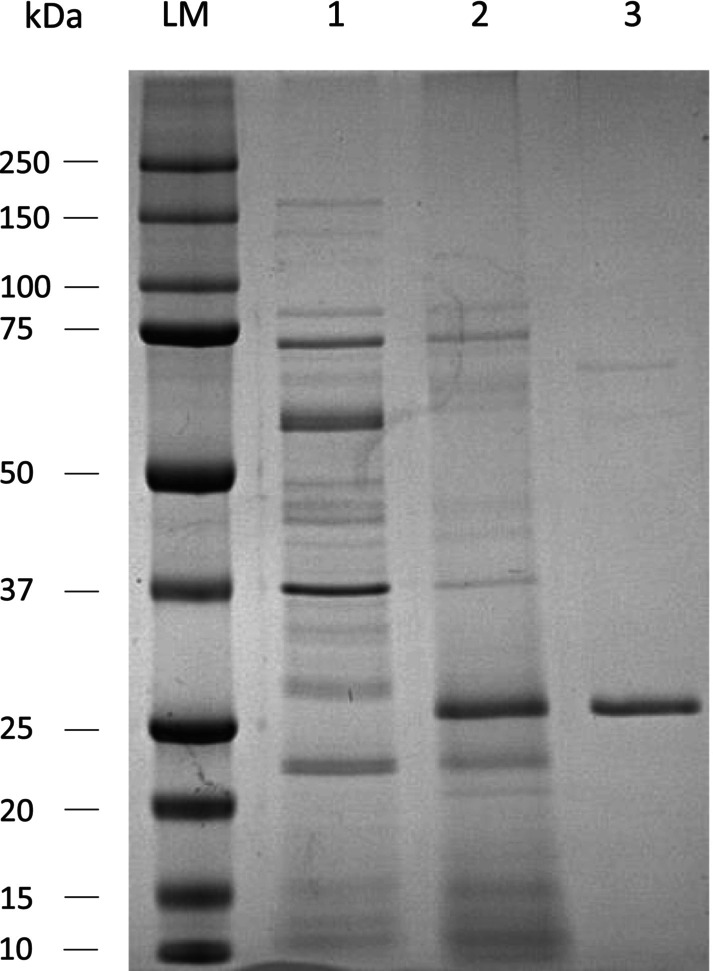
SDS/PAGE analysis of recombinant SPFA. Electrophoresis was performed using an 8–20% SDS polyacrylamide gel. Bio‐Rad Precision Plus Dual Color length marker (LM); culture supernatant of *B. subtilis* DB104 carrying pFF‐RED (1); culture supernatant of *B. subtilis* DB104 carrying pFF producing SPFA (2), after purification (3).

Production of recombinant SPFA by *B. subtilis* DB104 resulted in an activity of 32 U·mL^−1^ for suc‐AAPF‐pNA substrate and 418 U·mL^−1^ for azocasein in the supernatant. The purification to apparent homogeneity of SPFA was confirmed by SDS/PAGE (Fig. 3). The protease migrates at approximately 27 kDa, which is congruent with the theoretical molecular mass of 27.57 kDa. Furthermore, the molecular mass was confirmed to be 27.57 kDa by MALDI‐TOF MS analysis (Fig. S2). The purified SPFA had a specific activity of 195 U·mg^−1^ for the suc‐AAPF‐pNA substrate and 539 U·mg^−1^ for azocasein, respectively. The recovery yield was 5.92% with a purification fold of 2.98. Isoelectric point analysis of the purified and rebuffered protease revealed a pI of about 5.8, consistent with the predicted pI and an AB ratio of 0.9 with a high number of Asp residues (Fig. S3, Table S2).

### Effects of pH and temperature

**Fig. 4:**
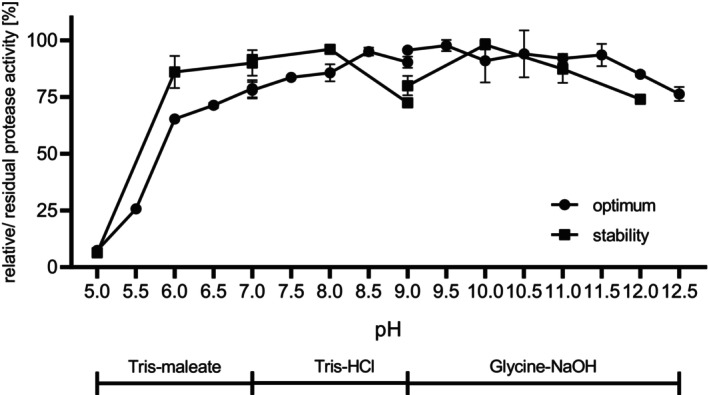
Influence of pH on the activity and stability of purified SPFA. Enzyme activity was determined using the suc‐AAPF‐pNA assay at 30 °C in a pH range of 5.0–12.5 (closed circles). The average maximum activity was considered as 100%: 64 U·mg^−1^. The effect of pH on the stability of purified SPFA (squares). The residual activity was measured with the standard suc‐AAPF‐pNA assay after incubation for 24 h at 4 °C in Tris‐maleate buffer (pH 5–7), in Tris–HCl (pH 7–9) and in glycine‐NaOH (pH 9–12). The activity at 0 h was considered as 100%; highest residual activity: 106 U·mg^−1^. Experiments were performed in triplicate, and data are presented as means ± SD.

The effects of pH on the activity of SPFA within the suc‐AAPF‐pNA assay were investigated at a pH range of 5.0–12.5 at 30 °C (Fig. 4). The protease showed a broad pH optimum between pH 8.5 and 11.5, while a relative activity of more than 75% was observed between pH 7.0 and 12.5. The relative activity at pH 5.0 and 12.0 was 8 and 76%, respectively. The stability test revealed a residual activity of at least 72% between pH 6.0 and 12.0, while SPFA was unstable at pH 5.0 (Fig. 4).

**Fig. 5:**
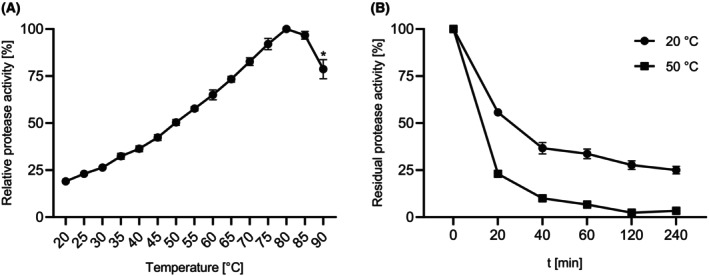
Effect of temperature on the activity (A) and stability (B) of purified SPFA. Enzyme activity was analysed at temperatures between 20 and 90 °C using the suc‐AAPF‐assay. The maximum activity was defined as 100%: 272 U·mg^−1^. * Enzyme stability did not last the intended 5 min. Stability was examined at 20 and 50 °C in 10 mm HEPES‐NaOH buffer, pH 8.0. Residual activity was measured using the suc‐AAPF pNA assay at 30 °C. The activity at 0 min was set at 100%: 85 U·mg^−1^. Experiments were performed in triplicate, and data are plotted as mean values ± SD.

The enzyme activity was investigated at pH 8.6 between 20 and 90 °C (Fig. 5A). The temperature optimum was reached at 80 °C and decreased to 79% residual activity at 90 °C. The temperature stability was investigated by incubating SPFA at 20 and 50 °C for 4 h (Fig. 5B). The activity of SPFA decreased to 25% after 4 h at 20 °C and to 3% at 50 °C. Possible autoproteolytic cleavage complicates the comparison of protease temperature stability. Hence, to monitor thermal protein unfolding rather than autoproteolysis, a thermal shift assay was conducted on SPFA. The protease was inhibited with phenylmethylsulfonyl fluoride (PMSF) and a denaturation curve with a melting point (*T*_m_) of 62.5 °C was obtained (Fig. S4).

### Effects of SDS, H_2_O_2_ and metal ions

The activity of SPFA after 1‐h incubation with 1% and 5% SDS (w/v) at 10 °C revealed high stability towards SDS, with increased activity after incubation with 1% and 5% SDS of 182% and 169%, respectively. SPFA showed a residual activity of 81% and 52% after 1 h of treatment with 1% and 5% H_2_O_2_ (v/v), respectively. Incubation of SPFA with 1 mm PMSF, a classical inhibitor for serine proteases [33], resulted in a complete inhibition. The effect of metal ions on protease activity after 1‐h incubation demonstrated residual activities of Mg^2+^ (94% ± 1%), Zn^2+^ (83% ± 1%), Mn^2+^ (95% ± 2%), Ca^2+^ (101% ± 0%), Co^2+^ (93% ± 1%), Ni^2+^ (90% ± 1%), Fe^2+^ (96% ± 1%) and Cu^2+^ (89% ± 1%). The incubation with EDTA showed no decrease in enzyme activity at 20 mm concentration.

### Effects of NaCl and proteolytic activity on synthetic peptides

**Fig. 6:**
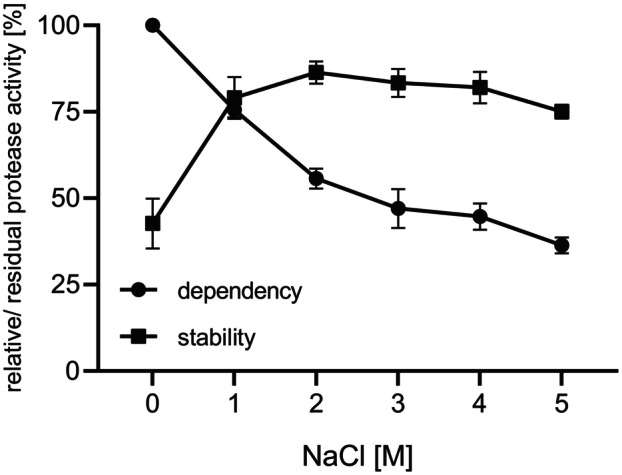
Activity and stability of purified SPFA at different NaCl concentrations. Activity was measured with the suc‐AAPF‐pNA assay in standard buffer (pH 8.6) at 30 °C with different NaCl concentrations (0–5 m). Maximum activity was defined as 100%: 104 U·mg^−1^. Stability was tested in 10 mm HEPES‐NaOH buffer, pH 8.0, with NaCl (0–5 m). The residual activity was measured with the suc‐AAPF‐pNA assay in standard buffer at pH 8.6 after incubation for 2 h at 20 °C. The activity before incubation for each NaCl concentration was defined as 100%. The experiments were performed in triplicate, and data are displayed as means ± SD.

Examination of the effect of different NaCl concentrations (0–5 m) in the suc‐AAPF‐pNA assay showed that SPFA reached maximum activity without NaCl and the activity declined gradually with higher NaCl levels to 36% at 5 m NaCl (Fig. 6). In contrast, the lowest stability was observed without NaCl with a residual activity of 43%, while the stability at 1–5 m NaCl was above 75%.

Against the synthetic peptide 4‐nitroanilide substrates, SPFA exhibited very low specificity for suc‐TVAA‐pNA, suc‐YVAD‐pNA, suc‐AAA‐pNA and suc‐AAVA‐pNA. The highest activity was shown for suc‐ALPF‐pNA and suc‐AGPP‐pNA (Table 1).

**Table 1:** Substrate specificities of SPFA against 10 synthetic substrates (suc‐XXXX‐pNA). Experiments were performed for 5 min at 30 °C with 17 mm substrate in 0.1 m Tris–HCl‐Puffer pH 8.6 with 0.1% (w/v) Brij®35. The experiment was executed in triplicates with a standard deviation of <5%. The enzyme activity against AAPF was defined as 100%.

Protease	Relative activity [%]									
	FAAF	AAA	AAVA	ALPF	AGPF	AAPF	TVAA	YVAD	AGPP	AAPL
SPFA	39	1	3	151	98	100 (117 U·mg^−1^)	3	1	181	81
Subtilisin Carlsberg^a^	57	0	2	60	90	100 (570 U·mg^−1^)	1	1	147	104
Savinase^a^	605	8	22	117	96	100 (180 U·mg^−1^)	5	5	144	12
BPN'^a^	96	0	6	106	96	100 (181 U·mg^−1^)	0	0	61	67

^a^ Falkenberg *et al*. [24].

## Discussion

Serine peptidases, especially the group of subtilisins from the subtilase family, are extremely versatile and of specific interest for biotechnological applications due to their properties such as broad pH range, high specificity and thermostability [12]. The *Bacillaceae* family has been recognised as a valuable source of subtilisins with high potential for industrial applications [2]. In this study, we therefore characterised the subtilisin SPFA from *Fictibacillus arsenicus* DSM 15822^T^, which was found through a data mining‐based search, as we previously published [6].

The comparison of the mature SPFA amino acid sequence to the well‐characterised true subtilisins Carlsberg [34], BPN’ [35] and subtilisin DY [36] reveals a sequence identity of 61.8%, 61.3% and 71.9%, respectively. When comparing SPFA amino acid sequence to our previously reported true subtilisins from halophilic and halotolerant background a sequence identity of 64.2% (SPMI), 65.4% (SPPM) and 71.7% (SPLA) was reached [27]. A more distant relationship exists to the high‐alkaline subtilisin Savinase from *Lederbergia lenta* (formerly *Bacillus lentus*; 52.2%) [37] and our previously reported high‐alkaline subtilisin SPAO from *Halalkalibacter okhensis* K10‐101^T^ (47.8%) [24], as well as the PIS SPAH (48.1%) from *Alkalibacillus haloalkaliphilus* DSM 5271^T^ [27]. The comparison of the amino acid sequences in the MSA confirms that SPFA belongs to the group of true subtilisins. Here, compared with BPN’, SPFA did not exhibit an amino acid deletion around position 160 that high‐alkaline subtilisins have in common [6]. Additionally, insertions compared with BPN' that are common for PIS cannot be observed [6]. Furthermore, SPFA displayed a molecular mass of 27.57 kDa, which is typical for subtilisins found from *Bacillaceae* used in the detergent industry [38].

The classification of SPFA as a member of the true subtilisin subgroup is especially remarkable because high activity at alkaline pH of 12.0 is usually subjected to members of the high‐alkaline subgroup [12]. Another example is the phylogenetically intermediate subtilisin ALTP from *Alkaliphilus transvaalensis*, which showed increasing activity up to the optimum of pH 12.0 [39]. SPFA is active and stable over a broad pH range (6.0–12.5) with a broad pH optimum. This broad pH optimum is quite unusual, while a broad pH stability is common to subtilases [40, 41, 42]. Tekin *et al*. [43] reported on the high‐alkaline subtilisin aprM from *Halalkalibacterium halodurans* C‐125 that also displays a high activity (> 70%) between pH 7.0 and 12.0 but with a clear optimum at pH 12.0. We also recently reported on three true subtilisins and one PIS derived from halotolerant and halophilic *Bacillaceae* that exhibited good activity even at a pH of 12.0, probably due to the salt adaptation through a charged molecular surface that facilitates the adaptation to high pH [27]. This charged molecular surface can also be observed in SPFA. However, especially on the back of the active site, positive and negative charges are almost evenly distributed. The fact that SPFA is still active at high NaCl concentrations is likely due to the charged amino acids on the protein surface, which form a hydrate shell around the protein and thus maintain its solubility. Here, negatively charged amino acids are the most favourable, followed by positively charged and charge‐neutral amino acids [44]. The increasing stability at higher NaCl concentrations is most likely due to the reduced activity and thus lower autoproteolysis.

Furthermore, the high temperature optimum of 80 °C is particularly noteworthy since *F. arsenicus*, the microbial origin of SPFA, is only able to grow below 40 °C [20]. Higher catalytic activity than the optimal growth temperature is common to enzymes originating from mesophiles (15–50 °C) [45]. Other subtilisins such as BPN’, Savinase and subtilisin Carlsberg displayed lower optimal temperatures (between 55 and 65 °C) than SPFA [24]. Similar to SPFA, the subtilase Aqualysin I from thermophilic *Thermus aquaticus* YT 1 also showed a high temperature optimum of 75–90 °C [46]. The phylogenetically intermediate subtilisin ALTP from *Alkaliphilus transvaalensis* exhibited a temperature optimum of 70 °C [39].

The stability towards SDS and H_2_O_2_ is interesting for industrial applications such as detergents. Here, SPFA showed increased activity when incubated with SDS, which has also been observed in other subtilisins [47, 48, 49]. In contrast, the two subtilisins from *Bacillus mojavensis* A21 or the subtilisin from *Bacillus pumilus* BO1 lost their activity when incubated with 1% (w/v) SDS [50, 51]. In terms of stability against oxidation with H_2_O_2_, SPFA showed good stability and retained more than half of its activity when incubated with 5% (v/v) H_2_O_2_. The two *B. mojavensis* A21 subtilisins also showed comparable stability to SPFA, while subtilisin Carlsberg, BPN’ and Savinase lost more than two thirds of their activity [24, 50]. Even higher stability was observed for the subtilisin from *Bacillus safensis* RH12 or the alkaline protease from *Bacillus patagoniensis* at higher hydrogen peroxide concentrations [42, 52]. The sensitivity of proteases to oxidants is probably due to the oxidation of a conserved methionine near the catalytic site leading to inactivation [53]. However, it was also found that this oxidation can lead to an altered substrate spectrum instead of inactivation [54]. Metal ions like Mg^2+^, Mn^2+^, Co^2+^ and Fe^2+^ at a concentration of 1 mm after incubating SPFA for 1 h caused only a slight decrease in protease activity of up to 7%. On the contrary, Zn^2+^, Ni^2+^, Cu^2+^ reduced protease activity by up to 17%, while Ca^2+^ had no influence. However, the incubation with EDTA showed no decrease in activity. After incubation with Fe^2+^, Cu^2+^, Zn^2+^, Ca^2+^ and Mn^2+^, a subtilisin from *Bacillus pumilus* BO1 even displayed a slight increase of up to 13% in activity, while Co^2+^ reduced the residual activity to 81% and EDTA to 78% [51]. A higher decrease in activity after incubation with Ni^2+^, Cu^2+^ was observed for a subtilase from a *Bacillus cereus* strain, while the decrease after Co^2+^ incubation was comparable to that of SPFA [55]. The proteases of the S8 family are known to be calcium‐dependent and contain usually two binding sites [4, 56]. The observation that the addition of calcium had no influence on SPFA could be due to the fact that the two calcium binding sites are already sufficiently filled. However, even the addition of EDTA causes no decrease in activity. No effect of EDTA after incubating the serine protease from *B. clausii* GMBAE 42 was also reported by Kazan *et al*. [57] and by Raval *et al*. [58]. Kembhavi *et al*. demonstrated that EDTA for a protease from *Bacillus subtilis* only has a destabilising effect at higher temperatures [41].

The substrate spectrum of SPFA towards the different synthetic substrates exhibits a preference comparable to that of other subtilisins from the true subtilisin and PIS subgroup [24, 27]. SPFA demonstrated comparable specificity to the industrially relevant subtilisins Carlsberg and BPN’ which belong to the true subtilisins subgroup. Hence, the substrate specificity towards the synthetic substrate is less similar to Savinase, which belongs to the high‐alkaline subgroup.

In conclusion, this is the first report of cloning, production, purification and biochemical characterisation of the true subtilisin SPFA from *Fictibacillus arsenicus* DSM 15822^T^. With its substrate preference towards 10 synthetic peptide‐4‐nitroanilide substrates with three or four amino acids and its phylogenetic classification, SPFA can be assigned to the group of true subtilisins. Furthermore, SPFA displayed a temperature optimum of 80 °C and a very broad pH optimum between 8.5 and 11.5 with high activity (> 75%) in an extremely wide range between pH 6.0 and 12.5. SPFA is still active at NaCl concentrations up to 5 m and very stable against 5% (w/v) SDS and stable against 5% (v/v) H_2_O_2_. Due to its unique biochemical properties, SPFA has the potential for use in biotechnological applications.

## Conflict of interest

The authors declare no conflict of interest.

## Author contributions

FF, JB and PS conceived and designed the experiments. FF conducted the experiments, collected and analysed the data. SK purified SPFA and did the MALDI‐TOF MS experiment. FF wrote the original draft; FF, JB, MB and PS revised the manuscript. All authors contributed to the final manuscript. All authors read and approved the manuscript.

## Supporting information


**Fig. S1.** Homology model of the mature SPFA obtained using I‐TASSER software. *In silico* metal‐binding analysis predicted the existence of two Ca2 + −binding sites (yellow balls). The catalytic residues Asp32, His64 and Ser221 are shown in red.
**Fig. S2.** MALDI‐TOF mass spectra of SPFA. The labels on the peaks indicate the measured average molecular mass. The peaks correspond from right to left M/z up to M/5z.
**Fig. S3.** Determination of the pI of the purified proteases. Isoelectric focussing was performed with a SERVALYT™ PRECOTES™ wide range pH 3–10 precast gel according to the manufacturer's recommendations. Lane M, SERVA IEF marker 3–10; lanes 1 purified SPFA rebuffered in 10 mM HEPES‐NaOH pH 8.0.
**Fig. S4.** Normalised melting curve of purified SPFA. The melting point (Tm) at which 50% of the protein is unfolded (−) was determined using SYPRO® Orange as a fluorescent probe (Ex/Em = 470/550 nm) (5 x SYPRO® Orange, 10 mM HEPES‐NaOH pH 8.0, 3 mM PMSF). The experiment was performed in triplicates and data are plotted as mean values ± SD.
**Table S1.** Oligonucleotides for amplification of the gene for SPFA by PCR using genomic DNA of *F. arsenicus* as template.
**Table S2.** pI value and AB ratio calculation.

## Data Availability

The original contributions presented in this study are included in the article/Supplementary material, and further inquiries can be directed to the corresponding author.
