# Supplementary material for: Biochemical characterisation of a novel broad pH spectrum subtilisin from Fictibacillus arsenicus DSM 15822T
Source: FEBS Open Bio. 2023 Sep 7;13(11):2035–46. doi: 10.1002/2211-5463.13701 (PMC10626276; doi:10.1002/2211-5463.13701)
Supplement: Supplementary file 1 — Fig. S1. Homology model of the mature SPFA obtained using I‐TASSER software. In silico metal‐binding analysis predicted the existence of two Ca2 + −binding sites (yellow balls). The catalytic residues Asp32, His64 and Ser221 are shown in red. Fig. S2. MALDI‐TOF mass spectra of SPFA. The labels on the peaks indicate the measured average molecular mass. The peaks correspond from right to left M/z up to M/5z. Fig. S3. Determination of the pI of the purified proteases. Isoelectric focussing was performed with a SERVALYT™ PRECOTES™ wide range pH 3–10 precast gel according to the manufacturer's recommendations. Lane M, SERVA IEF marker 3–10; lanes 1 purified SPFA rebuffered in 10 mM HEPES‐NaOH pH 8.0. Fig. S4. Normalised melting curve of purified SPFA. The melting point (Tm) at which 50% of the protein is unfolded (−) was determined using SYPRO® Orange as a fluorescent probe (Ex/Em = 470/550 nm) (5 x SYPRO® Orange, 10 mM HEPES‐NaOH pH 8.0, 3 mM PMSF). The experiment was performed in triplicates and data are plotted as mean values ± SD. Table S1. Oligonucleotides for amplification of the gene for SPFA by PCR using genomic DNA of F. arsenicus as template. Table S2. pI value and AB ratio calculation. [file FEB4-13-2035-s001.pdf]

Title:

Biochemical characterisation of a novel broad pH spectrum subtilisin from *Fictibacillus arsenicus* DSM 15822^{T}

Author's names:

Fabian Falkenberg[1], Sophie Kohn[1], Michael Bott[2], Johannes Bongaerts[1], Petra Siegert[1]

Addresses:

1 Institute of Nano- and Biotechnologies, Aachen University of Applied Sciences, 52428, Jülich, Germany.
2 Institute of Bio- and Geosciences, IBG-1: Biotechnology, Forschungszentrum Jülich, 52425 Jülich, Germany.

Corresponding author:

P. Siegert, Institute of Nano- and Biotechnologies, Aachen University of Applied Sciences, 52428, Jülich, Germany
Tel.: +49 241 6009 53124
E-mail: siegert@fh-aachen.de
https://www.fh-aachen.de/forschung/inb

Supplementary

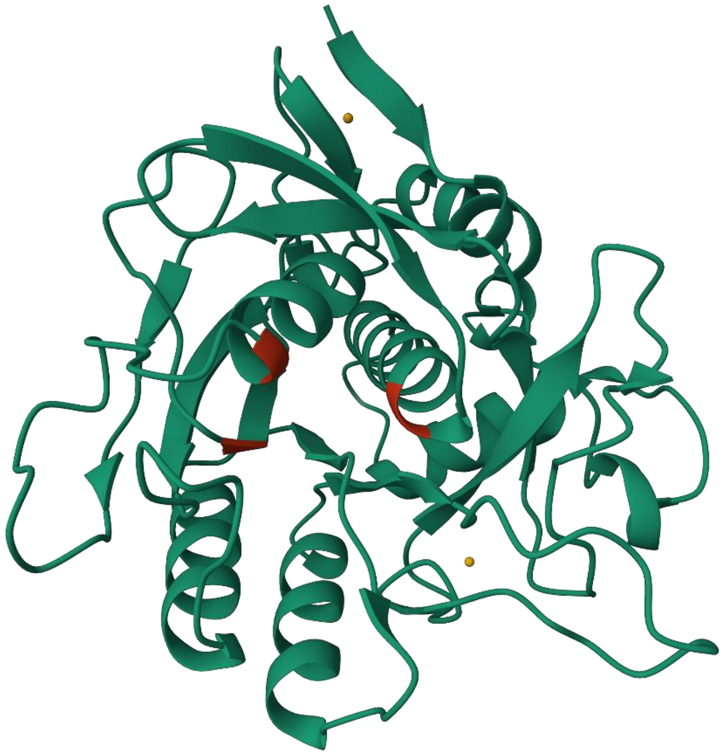

Fig. S1 Homology model of the mature SPFA obtained using I-TASSER software. *In silico* metal-binding analysis predicted the existence of two Ca^{2+}-binding sites (yellow balls). The catalytic residues Asp^{32}, His^{64}, and Ser^{221} are shown in red.

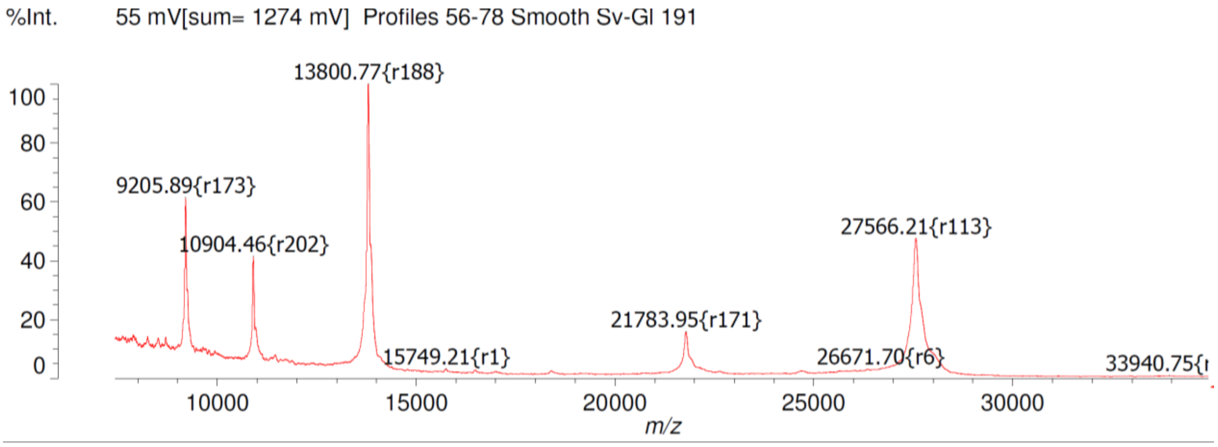

Fig. S2 MALDI-TOF mass spectra of SPFA. The labels on the peaks indicate the measured average molecular mass. The peaks correspond from right to left M/z up to M/5z.

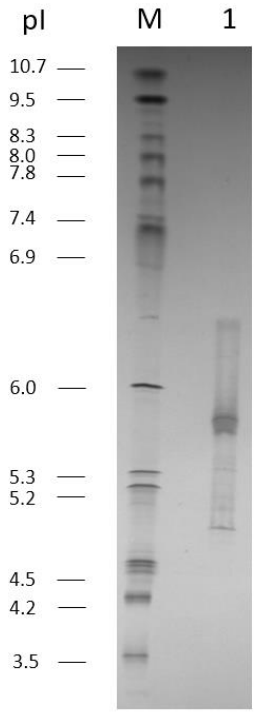

Fig. S3 Determination of the pI of the purified proteases. Isoelectric focusing was performed with a SERVALYT™ PRECOTES™ wide range pH 3-10 precast gel according to manufacturer recommendations. Lane M, SERVA IEF marker 3-10; lanes 1 purified SPFA rebuffered in 10 mM HEPES-NaOH pH 8.0.

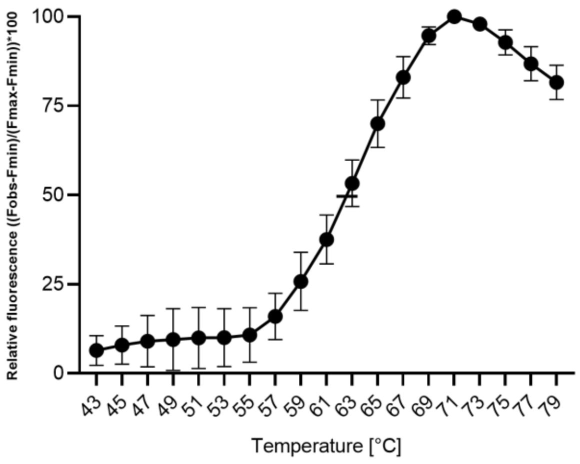

Fig. S4 Normalised melting curve of purified SPFA. The melting point (T_m) at which 50 % of the protein is unfolded (-) was determined using SYPRO® Orange as a fluorescent probe (Ex/Em = 470/550 nm) (5 x SYPRO® Orange, 10 mM HEPES-NaOH pH 8.0, 3 mM PMSF). The experiment was performed in triplicates and data are plotted as mean values ± SD.

Table S1 Oligonucleotides for amplification of the gene for SPFA by PCR using genomic DNA of *F. arsenicus* as template

Name	Sequence 5'->3'
aprE_F.arsenicus_fw	AGAAGACGAAATGAAAAAAACTGTATTACGCACG
aprE_F.arsencius_rv	AGAAGACATGTTATCTTGTCGCTGCGTAAAC

Table S2 pI value and AB ratio calculation

Protease	experimental pI	Number of residues					AB ratio[a]
		Arg	Asp	Glu	His	Lys	
SPFA	~5.8	5	13	4	6	8	0.9

[a] The AB ratio [AB = (Glu + Asp)/(Lys + His + Arg)] was calculated as described in (Rhodes et al. 2010).
